# Synchronization transitions caused by time-varying coupling functions

**DOI:** 10.1098/rsta.2019.0275

**Published:** 2019-10-28

**Authors:** Zeray Hagos, Tomislav Stankovski, Julian Newman, Tiago Pereira, Peter V. E. McClintock, Aneta Stefanovska

**Affiliations:** 1 Institute of Mathematical and Computer Sciences, University of São Paulo, São Carlos 13566-590, Brazil; 2 Department of Mathematics, Mekelle University, Mekelle, Ethiopia; 3 Faculty of Medicine, Ss Cyril and Methodius University, 50 Divizija 6, Skopje, North Macedonia; 4 Department of Physics, Lancaster University, Lancaster LA1 4YB, UK; 5 Department of Mathematics, Imperial College London, London SW7 2AZ, UK

**Keywords:** coupling functions, coupled oscillators, interactions, dynamical systems

## Abstract

Interacting dynamical systems are widespread in nature. The influence that one such system exerts on another is described by a coupling function; and the coupling functions extracted from the time-series of interacting dynamical systems are often found to be time-varying. Although much effort has been devoted to the analysis of coupling functions, the influence of time-variability on the associated dynamics remains largely unexplored. Motivated especially by coupling functions in biology, including the cardiorespiratory and neural delta-alpha coupling functions, this paper offers a contribution to the understanding of effects due to time-varying interactions. Through both numerics and mathematically rigorous theoretical consideration, we show that for time-variable coupling functions with time-independent net coupling strength, transitions into and out of phase- synchronization can occur, even though the frozen coupling functions determine phase-synchronization solely by virtue of their net coupling strength. Thus the information about interactions provided by the shape of coupling functions plays a greater role in determining behaviour when these coupling functions are time-variable.

This article is part of the theme issue ‘Coupling functions: dynamical interaction mechanisms in the physical, biological and social sciences’.

## 1. Introduction

Many dynamical systems, both natural and man-made, are composed of interacting parts. Examples include Josephson junctions [1,2], neuronal networks [3–5], the cardiorespiratory system [6–8], cardiorespiratory–brain interactions [9–12], and systems occurring in social sciences [13,14], communications [15,16] and chemistry [17–19]. Such systems often have external influences leading to time-variability in their mathematical description, e.g. time-varying frequency or a time-varying form of coupling function [20–24]. Dynamical systems whose evolution law is time-dependent, as opposed to temporally homogeneous, are said to be *non-autonomous* [25].

In a differential equation or stochastic differential equation describing a system of interacting components, the terms on the right-hand side arising from the interactions between the components are referred to as *coupling functions*. Coupling functions can be reconstructed from time series recorded from the interacting components, as a result of which one can obtain information about the interactions. For example, from cardiac and respiratory time series one can obtain the phase at which the cardiac beat is most susceptible to respiratory drive, from which one can extract the respiratory-related component of heart rate variability [26]. Another example is that general anaesthesia can lead to important changes in the forms of coupling function between brain waves [11]. Several studies show that the possession of time-varying coupling functions typifies the behaviour of interacting systems in real situations [27–29], and that such time-variations can play a major role in the dynamics [30–32].

The coupling function can be described by its net coupling strength, and its form [33]. By the net coupling strength, we mean the norm of the coupling function; its form, on the other hand, defines the functional law specifying the interactions, and it thereby introduces a new dimension and perspective [28,29,33]. Thus the net coupling strength quantifies only one aspect of the coupling. Many recent studies of interactions are designed for, and focus exclusively on, the effect of the net coupling strength of interacting systems. This approach is often found in information-theoretic methods for the detection of directionality and causality of influence between time-series including, for example, methods for Granger causality, transfer entropy, mutual information and symbolic transfer entropy [34–37]. From this perspective, time-variability of coupling strength was observed in cardiorespiratory interaction in [36].

In this paper, we extend [23] by studying theoretically the effects of time-varying coupling functions that induce a transition to synchronization, while keeping the net coupling strength constant. In particular, we will analyse numerically a model of two coupled oscillators with time-varying coupling functions while maintaining a constant net coupling strength. We will thus obtain information about synchronization epochs and phase slips in terms of the dynamics of the phase difference of the oscillators. Then we will set out theoretical considerations and theorems for quantifying these phenomena. For all of this, we will consider unidirectional coupling, i.e. the master-slave configuration. We will demonstrate that fixing a constant value for the net coupling strength does not necessarily enable one to predict whether or not synchronization transitions will occur, even though for the same set-up in the absence of time-variability, the value of the net coupling strength would have been sufficient to determine synchronization.

The paper is organized as follows. To set the context and to provide some motivation for the study, §2 describes an experiment on the time-variability of biological interactions. Section 3 presents a typical model of two coupled phase oscillators. Some basic concepts regarding coupling functions are presented in §4. Section 5 is devoted to the numerical simulations of the model. Theoretical consideration of the results, together with theorems formalizing these considerations, and their proofs, are given in §6. Discussion of connections to related work is presented in §7. Finally, we draw conclusions in §8.

## 2. Motivation from time-variability of biological interactions

Much of the motivation for studying time-varying coupling functions has come from biology. In particular, numerous situations arise where there are inherent time-variations, not only in the internal parameters and the quantitative characteristics of components of the system but also in the physical laws and functions that define the interactions between these components.

For example, the coupling functions of cardiorespiratory interactions were found to vary in time in [27], where it was shown that not only the coupling strength but also the form of the coupling function varies over time. Similarly, human delta-alpha neural coupling functions in the resting state also vary in time [28]. To illustrate, we now consider an example of time-varying cardiorespiratory and delta-alpha neural coupling functions, calculated using simultaneous recordings from the same subject. More specifically, the cardiorespiratory interactions were analysed from an ECG signal and a respiration-belt signal, while the delta and alpha brainwaves were extracted from an EEG signal, measured on the left forehead (equivalent to an FP1 electrode in the 10-20 EEG standard). These particular relationships were chosen for analysis, as they had previously been found significant when tested against surrogates. During recordings, the subject was resting in a supine position. The data are drawn from an earlier study of the effects of general anaesthesia on physiological oscillations [11]. Here, in order to demonstrate the time-variability properties, we consider data from only one of the earlier subjects.

Coupling functions were extracted from the phase dynamics using the dynamical Bayesian inference method [27,38,39], which involves the use of a sliding time-window to reveal the time-dependence. It was assumed that within each time-window, the phases *ϕ*_1_(*t*) and *ϕ*_2_(*t*) of the two interacting oscillators are governed by coupled differential equations of the general form (3.3, see §3 below) with the addition of Gaussian white noise, i.e.

$${\dot{\phi }}_{1}=\omega_{1}+q_{1}(\phi_{1},\phi_{2})+\xi_{1}(t)$$

and

$${\dot{\phi }}_{2}=\omega_{2}+q_{2}(\phi_{1},\phi_{2})+\xi_{2}(t),$$

where *ω*_*i*_ and *ϕ*_*i*_ are the natural frequency and phase of oscillator *i*, *ξ*_*i*_(*t*) is Gaussian white noise, and *q*_*i*_ is the coupling function describing the influence of oscillator *j* on the phase of oscillator *i*. First, from the recorded time-series the phases of the cardiac *ϕ*_*h*_, the respiratory *ϕ*_*r*_, the delta brainwave *ϕ*_*δ*_ and the alpha brainwave *ϕ*_*α*_ oscillations were extracted using the Hilbert protophase-to-phase procedure [40]. The coupling functions were then inferred within each time-window up to a second order of Fourier expansion on the 2-torus. In this way, we inferred the time-evolving cardiorespiratory coupling function *q*_*h*_(*ϕ*_*r*_, *ϕ*_*h*_) for the influence of the respiration on the heart, and the time-evolving neural cross-frequency coupling function *q*_*α*_(*ϕ*_*δ*_, *ϕ*_*α*_) for the influence of *δ* brainwaves on *α* brainwaves.

**Figure 1.**
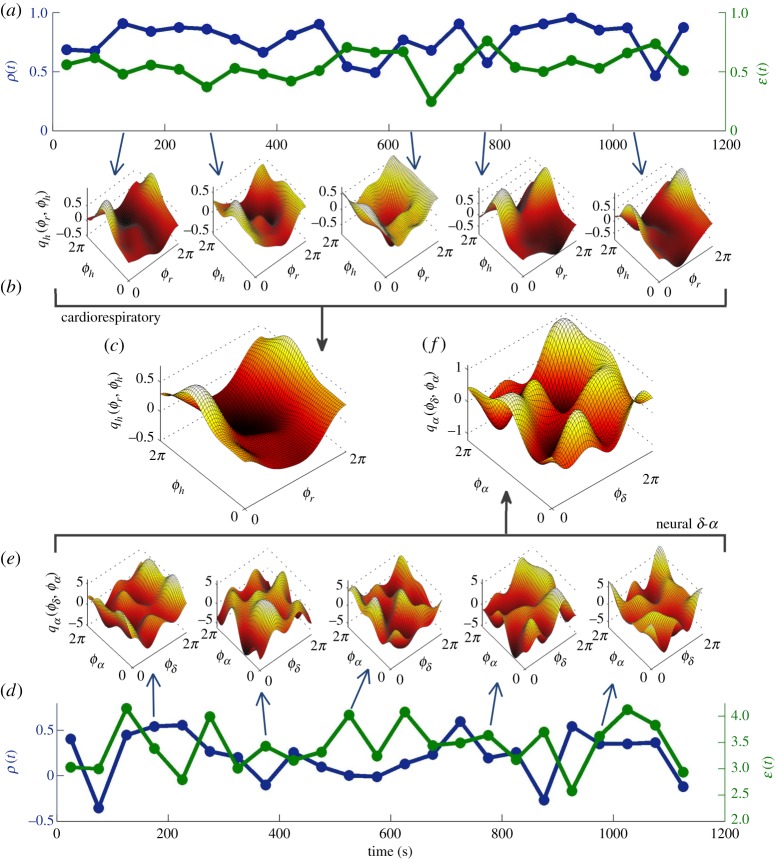
Time-variability of cardiorespiratory and delta-alpha neural interactions. Here, panels (*a*–*c*) show results of phase coupling from respiration to cardiac oscillations, while panels (*d*–*f* ) show results of phase coupling from delta brainwaves to alpha brainwaves. Panel (*a*) plots the time-variability of the similarity of form of coupling functions *ρ*(*t*) (blue line, left ordinate) and the net coupling strength *ε*(*t*) (green line, right ordinate) for the cardiorespiratory interactions. The similarity index *ρ*(*t*) is calculated with respect to the time-averaged coupling function. The five plots in (*b*) show the changes in the cardiorespiratory coupling function at different times; the time of each is indicated by a small arrow from the time axis in (*a*). For comparison, Panel (*c*) presents the time-averaged cardiorespiratory coupling function. Panels (*d*–*f* ) follow the same logic of presentation, but for delta-alpha neural coupling functions.

Figure 1 shows the results for cardiorespiratory interactions in (*a*–*c*) and for interactions in the neural delta and alpha waves in (*d*–*f* ). We show the time-variations in quantitative measures of the coupling functions (figure 1*a*), namely the coupling strength *ε*(*t*), and the similarity index *ρ*(*t*)∈[ − 1, 1] between the *form* of the coupling function and the form of the time-averaged coupling function. The coupling strength *ε*(*t*) is the norm (with respect to *L*^2^) of the coupling function at time *t*, as described in §4a; the similarity index *ρ*(*t*) [26,29,33,41] is the cosine similarity (again with respect to *L*^2^) between the coupling function at time *t* and the time-averaged coupling function. Note that both *ε*(*t*) and *ρ*(*t*) are time-varying, but they often vary differently, reinforcing the argument that the strength and form of the coupling represent two separate dimensions of the coupling function, often carrying different information about the interactions. By observing the time-variability of the cardiorespiratory coupling functions across different time windows (figure 1*b*) it is evident that there are smaller or larger variations in the form of the function when compared, for example, to the time-averaged coupling function (figure 1*c*).

Similarly, the delta-alpha neural coupling functions exhibit variations in form over time. Namely, the quantitative measures *ε*(*t*) and *ρ*(*t*) in figure 1*d* show different and even greater variability. The coupling functions calculated in different windows (figure 1*e*) vary abruptly and are not very similar to the time-averaged coupling function (figure 1*f* ). It seems possible that even greater variations of the separate coupling functions may have occurred within individual time windows, but that these variations were largely averaged out.

Comparing the cardiorespiratory and neural interactions shows that the former exhibits the more stable and invariant form, in that it varies less between different time windows, arguably implying greater determinism as described in the time-averaged coupling function. On the other hand, the neural coupling functions vary more and are individually less similar to the time-averaged coupling function. (These findings are consistent with previous results for the resting state in the multi-subject studies [11]). Common to both of the interactions is that they are time-varying, to a lesser or greater extent, and that the coupling strength and the form of the function often vary over time quite differently from each other. Hence, these characteristics can have correspondingly different effects on the outcome and the possible transitions caused by the interactions—a phenomenon worth exploring further theoretically.

## 3. Model

Rhythmic phenomena can be described by a stable periodic dynamics. Consider two weakly coupled oscillators described by the dynamical system

3.1 $$\begin{array}{rl} \frac{dx_{1}}{dt} & =f_{1}(x_{1})+\varepsilon g_{1}(x_{1},x_{2})\\ \;\mathrm{and}\;\frac{dx_{1}}{dt} & =f_{2}(x_{2})+\varepsilon g_{2}(x_{1},x_{2}), \end{array}$$

where *f*_1,2_ represent the unperturbed dynamics of the first and second oscillators, *g*_1,2_ represent the effects of one oscillator on the other and *ε* is a small parameter which represents the strength of the perturbations. We assume that *f*_*i*_ has an exponentially stable limit cycle *γ*_*i*_ of period *T*_*i*_ for *i* = 1, 2; and that the phase of the *i*th oscillator *ϕ*_*i*_ is defined on the limit cycle *γ*_*i*_ in such a way that it grows monotonically with time, satisfying the phase equation

3.2 $$\frac{d\phi_{i}}{dt}=\omega_{i},$$

for *i* = 1, 2, where *ω*_*i*_ = 2*π*/*T*_*i*_ is the natural frequency of the *i*th oscillator.

One can apply the phase reduction method to reduce the dynamics of the high-dimensional system equation (3.1) to lower-dimensional phase equations [42–46]. Small external perturbations to each oscillator *x*_*i*_, such as its interaction with the other oscillator *x*_*j*_, may force *x*_*i*_ off the limit cycle *γ*_*i*_ of *f*_*i*_. Therefore, for phase reduction of equation (3.1), one needs to define the phases outside the limit cycles. Using the concept of ‘isochrons’ [43,47,48] which are the level sets of phases, one can extend the definitions of the phases *ϕ*_1_ and *ϕ*_2_ of the oscillators to the whole basins of the corresponding limit cycles *γ*_1_ and *γ*_2_ in such a way that they rotate uniformly according to equation (3.2), not only on the cycle, but also in their corresponding neighbourhoods. With this, the model (3.2) of interacting oscillators can be reduced to a phase model taking the form

3.3 $$\begin{array}{rl} \frac{d\phi_{1}}{dt} & =\omega_{1}+q^{1}(\phi_{1},\phi_{2})\\ \;\mathrm{and}\;\frac{d\phi_{2}}{dt} & =\omega_{2}+q^{2}(\phi_{1},\phi_{2}), \end{array}$$

where the functions *q*^1^(*ϕ*_1_, *ϕ*_2_) and *q*^2^(*ϕ*_1_, *ϕ*_2_) are 2*π*-periodic with respect to their arguments. We refer to these functions *q*^1^ and *q*^2^ as the *coupling functions* for the phase-reduced model (3.3). When considering time-variable interactions, the coupling functions will be time-dependent functions *q*^1^_*t*_(*ϕ*_1_, *ϕ*_2_) and *q*^2^_*t*_(*ϕ*_1_, *ϕ*_2_).

In what follows, we will specifically consider *unidirectional* coupling, meaning that *q*^1^ = 0. But first, before we carry out our numerical and theoretical analysis of the model, we will in the next section introduce the concepts of net coupling strength and synchronization transitions.

## 4. Basic concepts

### (a) Net coupling strength

Because each coupling function *q* is 2*π*-periodic in both arguments, we can identify it on a torus of the form $T^{2}=S^{1}\times S^{1}$, where $S^{1}≅R/2\pi Z$. Hence, the torus $T^{2}$ can be identified by the square [ − *π*, *π*]^2^ or [0, 2*π*]^2^. We define the inner product in $L^{2}(T^{2},R)$ [49] by

$$\langle f,g\rangle =\frac{1}{4\pi^{2}}\int_{-\pi }^{\pi }\int_{-\pi }^{\pi }f(\phi_{1},\phi_{2})g(\phi_{1},\phi_{2})\;d\phi_{1}\;d\phi_{2}\text{ and the norm }∥f{∥}_{2}^{2}=\langle f,f\rangle .$$

If the coupling function *q* is smooth, then we can decompose *q* into a Fourier series on the square [ − *π*, *π*]^2^, using Parseval's identity [49] to compute the norm in terms of Fourier coefficients.

In applications, the coupling functions can be well approximated by using a finite number of Fourier terms. Each coupling function is typically of the diffusive type

4.1 $$\begin{array}{rl} q_{t}(\phi_{1},\phi_{2}) & =c_{1}(t)\sin(\phi_{1}-\phi_{2})+c_{2}(t)\cos(\phi_{1}-\phi_{2})\\ & =(c_{1}(t)+c_{2}(t))\sin(\phi_{1})\cos(\phi_{2})+(c_{2}(t)-c_{1}(t))\cos(\phi_{1})\sin(\phi_{2}), \end{array}$$

where *c*_1_(*t*) and *c*_2_(*t*) are time-varying parameters. With the second expression for *q*_*t*_ in equation (4.1) being a finite sum of Fourier components, Parseval's identity gives

4.2 $$∥q_{t}{∥}_{2}=\frac{1}{\sqrt{2}}\sqrt{c_{1}^{2}(t)+c_{2}^{2}(t)}.$$

This norm provides a quantitative measure of the net coupling strength between the linked systems. For any specified (time-dependent) form of coupling function, this norm represents the scaling parameter of the coupling function.

### (b) Intermittent synchronization

**Figure 2.**
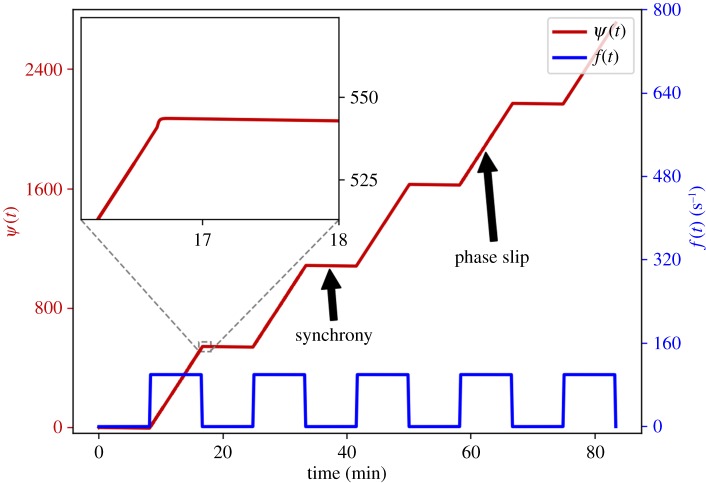
Synchronization transitions in the model equation (5.1), due to a time-varying coupling function *q*_*t*_ in equation (4.1). Specifically, $c_{1}(t)=\sqrt{2}\alpha \cos(f(t)t)$ and $c_{2}(t)=\sqrt{2}\alpha \sin(f(t)t)$ as in equation (5.3), where *f*(*t*) is the periodic function defined in equation (5.4). In red is shown the phase difference *ψ*(*t*) = *ϕ*_1_(*t*) − *ϕ*_2_(*t*) as governed by equation (5.2), and in blue is shown *f*(*t*). The parameters *ε* and *k* were set to *ε* = 0.01 rad s^−1^, *k* = 100 rad s^−1^, and the net coupling strength was set to $\alpha =1.55/\sqrt{2}\;s^{-1}$. The inset shows the transition to synchronization. The dynamics of the phase difference is shown to alternate between synchrony states and phase slips (indicated by bold arrows in the plot of *ψ*(*t*)), due to the time-variability of the coupling function *q*_*t*_ in equation (4.1) via the parameters *c*_1_(*t*) and *c*_2_(*t*) while the net coupling strength remains constant.

**Figure 3.**
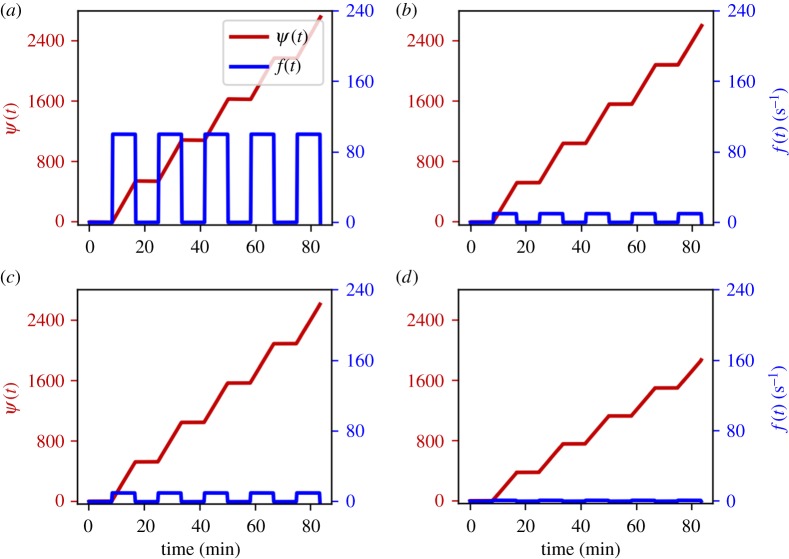
Synchronization transitions due to a time-varying coupling function, like in figure 2, with different values of the parameters *ε* and *k* for the function *f*(*t*) in equation (5.4). In all four plots, in red is shown the phase difference *ψ*(*t*), and in blue is shown *f*(*t*). The parameters *ε* and *k* were set to (*a*) *ε* = 0.01, *k* = 100, (*b*) *ε* = 0.001, *k* = 10, (*c*) *ε* = 0.0001, *k* = 10 and (*d*) *ε* = 0, *k* = 1 (all in rad s^−1^). In all four plots, the net coupling strength was set to $\alpha =1.176/\sqrt{2}\;s^{-1}$. The plots of the phase difference reveal synchronous and asynchronous states due to the time-variability of the coupling function while the net coupling strength remains constant.

A mark of the level of adjustment of rhythmic behaviour due to interaction is whether it is sufficient to cause synchronization of the oscillators [42]. For time-variable coupling functions, the time-variability can cause transitions into or out of synchronization, i.e. it can cause there to be both epochs of synchrony during which the phase difference remains nearly constant and epochs of phase slipping during which the phase difference changes rapidly. An example to illustrate these behaviours is shown in figures 2 and 3 which plot time series of the phase difference between two coupled oscillators. We will sometimes refer to this behaviour as *intermittent synchronization*. We emphasize that these transitions into and out of synchronization are not the same as the intermittency of apparent synchronization often occurring in autonomous systems with parameters close to or on the boundary of a region of synchronization or chaos [42].

Transitions into and out of synchronization occurring due to time-variability of the coupling functions in equation (3.3) correspond physically to dynamical consequences of the *openness* of a coupled-oscillator system to time-variable external influences. An example of intermittent synchronization induced by such non-autonomicity of models of open systems is the focus of this present paper. It is also known that for closed systems, transitions into or out of synchronization can occur due to the time-evolution of slow variables within the system representing internal adaptation based on the current or previous state of the oscillators, as exemplified in [50] for a large network with slowly adaptive coupling via time-delays. The intermittency in this latter case is similar to intermittency induced by non-autonomicity, but represents a very different physical cause of the exhibited dynamical behaviour.

## 5. Numerics

Our main goal is to establish numerically the effect of time-variation in the coupling functions between two-phase oscillators. Our focus will be on the case where the coupling exists in one direction only, sometimes known as unidirectional coupling, or the master-slave configuration. We choose this configuration because it provides the clearest case where time-varying coupling functions can lead to synchrony and phase slips, while the net coupling strength remains constant.

Consider the master-slave configuration

5.1 $$\begin{array}{rl} \frac{d\phi_{1}}{dt} & =\omega_{1}\\ \;\mathrm{and}\;\frac{d\phi_{2}}{dt} & =\omega_{2}+q_{t}(\phi_{1},\phi_{2}), \end{array}$$

where the coupling function *q*_*t*_(*ϕ*_1_, *ϕ*_2_) is equal to the expression in equation (4.1) and *ω*_1_, *ω*_2_ are the natural frequencies of the oscillators. The presence of the coupling term *q*_*t*_(*ϕ*_1_, *ϕ*_2_) could cause the fundamental frequency of the driven oscillator (whose phase is represented by *ϕ*_2_(*t*)) to become different from its natural frequency *ω*_2_, and to become time-dependent as *q*_*t*_ varies over time.

From the previous section, the net coupling strength of the master-slave configuration *q*_*t*_ in equation (5.1) can be defined as

$$∥q_{t}{∥}_{2}=\alpha (t)=\frac{1}{\sqrt{2}}\sqrt{c_{1}^{2}(t)+c_{2}^{2}(t)},$$

where *c*_1_(*t*) and *c*_2_(*t*) are the time-varying coupling parameters of the coupling functions [23]. In the autonomous case, where the coupling parameters *c*_1_ and *c*_2_ are constant, if the net coupling strength $∥q{∥}_{2}=\alpha >\frac{1}{\sqrt{2}}|\omega_{1}-\omega_{2}|$ then the oscillators will synchronize [23]. In the present study, unlike the autonomous case, the oscillators are not guaranteed to be synchronized all of the time. With constant net coupling strength $∥q_{t}{∥}_{2}=\alpha >\frac{1}{\sqrt{2}}|\omega_{1}-\omega_{2}|$, we will show that the oscillators can undergo transitions between synchrony and phase slipping.

To analyse whether the two oscillators synchronize or not, we consider the phase difference

$$\psi (t)=\phi_{1}(t)-\phi_{2}(t)$$

between them. From equation (5.1) with *q*_*t*_ as in equation (4.1), the phase difference *ψ*(*t*) obeys the equation

5.2 $$\frac{d\psi }{dt}=\Omega -c_{1}(t)\sin(\psi )-c_{2}(t)\cos(\psi ),$$

where *Ω* = *ω*_1_ − *ω*_2_ is the natural frequency difference (sometimes called the frequency mismatch, or detuning) between the oscillators. For numerical investigation of synchrony in our coupled oscillator model, we simulate the differential equation (5.2), taking the time-dependent coupling parameters *c*_1_(*t*) and *c*_2_(*t*) as

5.3 $$c_{1}(t)=\sqrt{2}\alpha \cos(f(t)t)\;\mathrm{and}\;c_{2}(t)=\sqrt{2}\alpha \sin(f(t)t),$$

where *f*(*t*) is a *T*-periodic function defined by

5.4 $$f(t)=\left\{ \begin{array}{ll} \varepsilon & 0\leq t\leq T_{1}\\ \frac{\varepsilon (T/2-t)+k(t-T_{1})}{T/2-T_{1}} & T_{1}\leq t\leq \frac{T}{2}\\ k & T/2\leq t\leq T_{2}\\ \frac{k(T-t)+\varepsilon (t-T_{2})}{T-T_{2}} & T_{2}\leq t\leq T \end{array}\right.$$

with *k* > *ε*≥0, and with the values of *ε*, (*T*/2 − *T*_1_)/*T* and (*T* − *T*_2_)/*T* being small. The expression for the function in equation (5.4) has been chosen to exhibit the existence of synchrony epochs and phase slips in the dynamics of the phase difference. Note that, from equation (5.3), we obtain

$$∥q_{t}{∥}_{2}=\alpha ,$$

showing that the net coupling strength is constant for all time.

In all our simulations, the length of the simulated time series is set to 5000 s and the sampling step is set as *h* = 0.001 s, and we use the following parameter values: *Ω* = 1.08 rad s^−1^, *T* = 1000 s, *T*_1_ = 490 s, *T*_2_ = 990 s. We will consider different possible values for the remaining parameters *k*, *ε* and *α*. We simulate equation (5.2) via its simplified form equation (6.1) with *φ*(*t*): = *f*(*t*)*t*, taking the initial value of the phase difference as *ψ*(0) = 0.

Figure 2 presents a time series of the phase difference *ψ*(*t*) (red) and the function *f*(*t*) (blue), and the zoomed figure (top-left inset) shows the transition to synchrony. Further numerical simulations were carried out using different parameter values, for which results are shown in figure 3. For both figures, the net coupling strength *α* is constant over time, with $\sqrt{2}\alpha$ being greater than the magnitude of the natural frequency difference *Ω*.

In both figures 2 and 3*a*–*d*, we see intermittency of synchronization, similar to that observed experimentally and numerically in [7,20,24,27,51]: there is an alternation between synchronized epochs (plateaux) and phase slips (rapid increases) in *ψ*(*t*). While *φ*(*t*): = *f*(*t*)*t* is slowly varying—i.e. on the intervals [*nT*, *nT* + *T*_1_] where *f*(*t*) has small magnitude—we have phase synchrony. But while *φ*(*t*) has rapid angular velocity—i.e. on the intervals $\left[(n+\frac{1}{2})T,nT+T_{2}\right]$ where *f*(*t*) is large—we have unbounded slipping of the phase difference.

Given that the net coupling strength between the systems was invariant, it is evident that the continuing alternation between synchronization epochs and phase slips was just due to time-variation in the coupling function. This shows that the net coupling strength does not in itself give us enough information to characterize the interactions of the oscillators. Based on the numerics implemented in this section, we can generalize the choices of the coupling parameters *c*_1_(*t*) and *c*_2_(*t*) in order to analyse the effects of the time-varying coupling functions.

## 6. Explanation and generalization of numerical findings

Consider equation (5.3) with a general function *φ*(*t*) in place of *f*(*t*)*t*. Based on its behaviour, we determine the dynamics of the phase difference *ψ*(*t*) of the oscillators. Whether they exhibit synchronous and/or asynchronous states hinges on three considerations:

(i) If *φ*(*t*) is a slowly varying function, i.e. $|\dot{\varphi }|$ is small, then the phase difference *ψ*(*t*) corresponds to a synchronous state for a finite time *T*_1_: for arbitrarily large *T*_1_ we can take $|\dot{\varphi }|$ sufficiently small that the solution will remain in an arbitrarily small ball. See theorem 6.1 and corollary 6.2 for the proof.
(ii) If the phase shift *φ*(*t*) is fast-winding round the circle (i.e. its unwrapped phase is rapidly growing) then, using an averaging argument, we can show that the dynamics induces phase slips. See theorem 6.3. The averaging argument essentially means that due to the fast timescale of *φ*, the oscillators *ϕ*_1_ and *ϕ*_2_ feel no interactions and they rotate independently at their own natural angular velocities.
(iii) If the function *φ*(*t*) has both the behaviours stated in (i) and (ii), then the coupled oscillators will synchronize when *φ* proceeds on the slow timescale, but they will exhibit phase drifts when *φ* proceeds on the fast timescale. Hence, the dynamics of the phase difference in equation (6.1) subject to a function *φ*(*t*) with both slow variation and fast winding induces both synchronous and asynchronous states.

We now provide rigorous theorems to justify these three considerations. Before doing so, however, we briefly discuss the setup. The phase difference *ψ*(*t*) = *ϕ*_1_(*t*) − *ϕ*_2_(*t*) obeys equation (5.2). We take the parameters *c*_1_(*t*) and *c*_2_(*t*) in equation (5.2) to be of the form

$$c_{1}(t)=\sqrt{2}\alpha \cos(\varphi (t))\;\mathrm{and}\;c_{2}(t)=\sqrt{2}\alpha \sin(\varphi (t)),$$

for some constant *α* > 0 and *C*^1^ function $\varphi :R\to S^{1}≅R/2\pi Z$. The constant *α* corresponds to the net coupling strength.

Equation (5.2) can be written as

6.1 $$\frac{d\psi }{dt}=\Omega -\sqrt{2}\alpha \sin(\psi +\varphi (t)).$$

Note that equation (6.1) is a non-autonomous system, and we can consider the effect of both slow variation and fast variation of *φ*(*t*). Introducing a new variable *η*(*t*) = *ψ*(*t*) + *φ*(*t*), the dynamics of equation (6.1) is then equivalent to the dynamics of the equation

6.2 $$\frac{d\eta }{dt}=\Omega +\dot{\varphi }(t)-\sqrt{2}\alpha \sin(\eta ).$$

In the case that $\sqrt{2}\alpha >|\Omega |$, let η* and η^**^ be respectively the stable and unstable fixed points of the equation

6.3 $$\frac{d\eta }{dt}=\Omega -\sqrt{2}\alpha \sin(\eta ).$$

The following theorems address the effect of the time-varying coupling functions, while the net coupling strength is invariant. First, in Theorem 6.1 and its subsequent corollary, we consider the synchronization exhibited for slowly varying *φ*.

**Theorem 6.1.**

*Assume* $\sqrt{2}\alpha >|\Omega |$. *For all* $\tilde{\varepsilon }>0$ *and* $\eta_{0}\in S^{1}\setminus \{\eta^{**}\}$*, there exist* *ε*_0_, *T*_0_ > 0 *such that for all* *t* > *T*_0_*, if* $|\dot{\varphi }|\leq \varepsilon_{0}$ *on* [0, *t*] *then the solution* *ψ* *of equation* (*6.1*) *with* *ψ*(0) = *η*_0_ − *φ*(0) *satisfies* $\psi (s)\in B_{\frac{1}{2}\tilde{\varepsilon }}(\eta^{*}-\varphi (s))$ *for all* *s*∈(*T*_0_, *t*].

**Proof.**

Fix $\tilde{\varepsilon }>0$ and $\eta_{0}\in S^{1}\setminus \{\eta^{**}\}$, assuming without loss of generality that *η*_0_ lies in the arc extending anticlockwise from η^**^ to η*, and that $\frac{1}{2}\tilde{\varepsilon }<\min(d(\eta^{*},\eta_{0}),d(\eta^{*},\pi /2)$). Thus the function $F(\eta ):=\Omega -\sqrt{2}\alpha \sin(\eta )$ is strictly positive on $\left[\eta_{0},\eta^{*}-\frac{1}{2}\tilde{\varepsilon }\right]$ and strictly negative at $\eta^{*}+\frac{1}{2}\tilde{\varepsilon }$. Accordingly, we can pick $\varepsilon_{0}<\min(F(\eta_{0}),F(\eta^{*}-\frac{1}{2}\tilde{\varepsilon }),-F(\eta^{*}+\frac{1}{2}\tilde{\varepsilon }))$ and let

$$T_{0}=\int_{\eta_{0}}^{\eta^{*}-(1/2)\tilde{\varepsilon }}\frac{1}{F(\eta )-\varepsilon_{0}}\;d\eta .$$

Then we have the following:

(i) If $|\dot{\varphi }|\leq \varepsilon_{0}$ on an interval [0, *t*], then $\Omega +\dot{\varphi }(s)-\sqrt{2}\alpha \sin(\eta )$ is positive at $\eta =\eta^{*}-\frac{1}{2}\tilde{\varepsilon }$ and negative at $\eta =\eta^{*}+\frac{1}{2}\tilde{\varepsilon }$ for all *s*∈[0, *t*]. Therefore, any solution *η*( · ) of equation (6.2) with $\eta (t_{0})\in \bar{B_{(1/2)\tilde{\varepsilon }}(\eta^{*})}$ for some *t*_0_∈[0, *t*) has $\eta (s)\in B_{(1/2)\tilde{\varepsilon }}(\eta^{*})$ for all *s*∈(*t*_0_, *t*].
(ii) If $|\dot{\varphi }|\leq \varepsilon_{0}$ on [0, *T*_0_], then there exists *t*_0_∈(0, *T*_0_] such that the solution *η*( · ) of equation (6.2) with *η*(0) = *η*_0_ has $\eta (t_{0})=\eta^{*}-\frac{1}{2}\tilde{\varepsilon }$.

Combining these gives the result. ▪

**Corollary 6.2.**

*Assume* $\sqrt{2}\alpha >|\Omega |$. *For all* $\tilde{\varepsilon }>0$ *and* $\eta_{0}\in S^{1}\setminus \{\eta^{**}\}$ *there exists* *T*_0_ > 0 *such that for all* *T*_1_ > *T*_0_*, there exists* *ε* > 0 *such that if* $|\dot{\varphi }|\leq \varepsilon$ *on* [0, *T*_1_] *then the solution* *ψ* *of equation* (*6.1*) *with* *ψ*(0) = *η*_0_ − *φ*(0) *satisfies* $\psi (t)\in B_{\tilde{\varepsilon }}(\eta^{*}-\varphi (0))$ *for all* *t*∈(*T*_0_, *T*_1_].

**Proof.**

Take *ε*_0_ and *T*_0_ as in Theorem 6.1, and then for *T*_1_ > *T*_0_ take $\varepsilon =\min(\varepsilon_{0},\frac{\tilde{\varepsilon }}{2T_{1}}$). So if $|\dot{\varphi }|\leq \varepsilon$ on [0, *T*_1_] then $d(\varphi (t),\varphi (0))\leq \frac{1}{2}\tilde{\varepsilon }$ for all *t*∈[0, *T*_1_], and so we have the desired result. ▪

Now in Theorem 6.3, we consider the unbounded phase slips exhibited for fast-winding *φ*. The following theorem may be regarded as a kind of non-autonomous averaging principle. For a detailed exposition of averaging principles, see [52].

**Theorem 6.3.**

*For all* ${\tilde{\varepsilon }}_{0},\tilde{\varepsilon }>0$ *there exist* *K*_1_, *K*_2_ > 0 *such that if* *φ* *is twice differentiable on an interval* [0, *t*] *with* $\dot{\varphi }(s)>\max(K_{1},K_{2}\sqrt{|\ddot{\varphi }(s)|})$ *for all* *s*∈[0, *t*]*, then any solution* *ψ* *of equation* (*6.1*) *has*

$$|\psi (s)-\psi (0)-\Omega s|\leq {\tilde{\varepsilon }}_{0}+\tilde{\varepsilon }s$$

*for all* *s*∈[0, *t*].

**Proof.**

As in the proof of Theorem 6.1, let $F(\eta ):=\Omega -\sqrt{2}\alpha \sin(\eta )$. First take arbitrary *K*_1_ > 0 and $K_{2}>\sqrt{2\pi }$, and suppose that $\dot{\varphi }(s)>\max(K_{1},K_{2}\sqrt{|\ddot{\varphi }(s)|})$ for all *s*∈[0, *t*]. Define recursively a sequence 0 = *t*_0_ < *t*_1_ < · *s* < *t*_*N*_ < *t* by $t_{i+1}=t_{i}+2\pi /\dot{\varphi }(t_{i})$, with *N* being the largest possible such that *t*_*N*_ < *t*. It follows in particular that $t-t_{N}\leq 2\pi /\dot{\varphi }(t_{N})$. For each *i* < *N*, we have that

$$\int_{t_{i}}^{t_{i+1}}F\left(\psi (t_{i})+\varphi (t_{i})+\frac{2\pi (s-t_{i})}{t_{i+1}-t_{i}}\right)ds=\Omega (t_{i+1}-t_{i})$$

and therefore

6.4 $$\begin{array}{rl} & |\psi (t_{i+1})-\psi (t_{i})-\Omega (t_{i+1}-t_{i})|\\ & \;\leq \int_{t_{i}}^{t_{i+1}}\left|F(\psi (s)+\varphi (s))-F\left(\psi (t_{i})+\varphi (t_{i})+\frac{2\pi (s-t_{i})}{t_{i+1}-t_{i}}\right)\right|ds. \end{array}$$

Now fix any *s*∈(*t*_*i*_, *t*_*i*+1_). We have that *s* − *t*_*i*_ < 2*π*/*K*_1_ and so

6.5 $$|\psi (s)-\psi (t_{i})|<\frac{2\pi (|\Omega |+\sqrt{2}\alpha )}{K_{1}}.$$

Also, by Taylor's theorem, we have that

$$\left|\varphi (s)-\left(\varphi (t_{i})+\frac{2\pi (s-t_{i})}{t_{i+1}-t_{i}}\right)\right|=\frac{1}{2}|\ddot{\varphi }(\xi_{1}(s))|{\left(s-t_{i}\right)}^{2}\leq \frac{2\pi^{2}|\ddot{\varphi }(\xi_{1}(s))|}{\dot{\varphi }{\left(t_{i}\right)}^{2}}$$

for some *ξ*_1_(*s*)∈(*t*_*i*_, *s*). But by the mean value theorem we have that for some *ξ*_2_(*s*)∈(*t*_*i*_, *ξ*_1_(*s*)),

$$\frac{1}{\dot{\varphi }(\xi_{1}(s))}=\frac{1}{\dot{\varphi }(t_{i})}-\frac{\left(\xi_{1}(s)-t_{i})\ddot{\varphi }(\xi_{2}(s)\right)}{\dot{\varphi }{\left(\xi_{2}(s)\right)}^{2}}>\frac{1}{\dot{\varphi }(t_{i})}\left(1-\frac{2\pi }{K_{2}^{2}}\right)$$

and so

6.6 $$\left|\varphi (s)-\left(\varphi (t_{i})+\frac{2\pi (s-t_{i})}{t_{i+1}-t_{i}}\right)\right|\leq \frac{2\pi^{2}|\ddot{\varphi }(\xi_{1}(s))|}{\dot{\varphi }{\left(\xi_{1}(s)\right)}^{2}}{\left(1-\frac{2\pi }{K_{2}^{2}}\right)}^{-2}\leq \frac{2\pi^{2}}{K_{2}^{2}}{\left(1-\frac{2\pi }{K_{2}^{2}}\right)}^{-2}.$$

Combining equations (6.4), (6.5) and (6.6), we have that

$$|\psi (t_{i+1})-\psi (t_{i})-\Omega (t_{i+1}-t_{i})|\leq \underset{=:\kappa (K_{1},K_{2})}{\underbrace{\sqrt{2}\alpha \left(\frac{2\pi (|\Omega |+\sqrt{2}\alpha )}{K_{1}}+\frac{2\pi^{2}}{K_{2}^{2}}{\left(1-\frac{2\pi }{K_{2}^{2}}\right)}^{-2}\right)}}(t_{i+1}-t_{i}).$$

Hence, for each *i* ≤ *N*,

6.7 $$|\psi (t_{i})-\psi (0)-\Omega t_{i}|\leq \kappa (K_{1},K_{2})t_{i}.$$

Now for any *s*∈[0, *t*], taking the largest *i* with *t*_*i*_ ≤ *s*, we have

$$|\dot{\psi }(\zeta )-\Omega |\leq \sqrt{2}\alpha \;\forall \zeta \in [t_{i},s]$$

and so

6.8 $$|\psi (s)-\psi (t_{i})-\Omega (s-t_{i})|\leq \sqrt{2}\alpha (s-t_{i})<\underset{=:\kappa_{0}(K_{1})}{\underbrace{\frac{2\sqrt{2}\pi \alpha }{K_{1}}}}.$$

Combining equations (6.7) and (6.8) gives that

$$|\psi (s)-\psi (0)-\Omega s|<\kappa_{0}(K_{1})+\kappa (K_{1},K_{2})s$$

for all *s*∈[0, *t*]. So now, given any ${\tilde{\varepsilon }}_{0},\tilde{\varepsilon }>0$ choose *K*_1_, *K*_2_ sufficiently large that $\kappa_{0}(K_{1})\leq {\tilde{\varepsilon }}_{0}$ and $\kappa (K_{1},K_{2})\leq \tilde{\varepsilon }$. ▪

Combining theorems 6.1 and 6.3, we have that if *φ* has alternating epochs of slow variation and rapid oscillation, this can lead to the system (5.1) having alternating epochs of synchrony and asynchrony.

## 7. The results in context

There are different ways in which time-variability can enter a system, and so other forms of non-autonomous driving that give rise to intermittent synchronization have also been considered. Of particular interest has been the case where time-variability enters through modulation of the natural frequency of the driving oscillator [22,24,53]. As shown in [24,53], for fixed-frequency driving we have synchronization either all of the time or none of the time, but when the driving frequency is allowed to vary then we can have intermittent synchronization. Through the finite-time dynamical considerations of [24] to account for a free shape of variation over time, it was shown that this intermittent synchronization results overall in stability of the driven oscillations, as indicated by negativity of Lyapunov exponents; and in this manner, greater time-variability of the driving frequency increases the region of stability in parameter space. Similar results were also observed numerically for higher-dimensional oscillatory systems.

The theoretical and numerical considerations of [24] for a unidirectionally coupled pair of phase oscillators have been extended to phase-oscillator networks in [54]. The considerations of this present paper can be generalized to networks such as those considered in [55]: network structure can also have an impact on the dynamics exhibited, such as synchronization [30,56]. Network synchronization induced by sufficiently fast non-autonomous driving has been considered in [57], where the non-autonomous driving consists of rapid switching between the existence and non-existence of links between given nodes, while the dynamical interaction along existing nodes takes a specific time-independent form.

As intermittent synchronization in [24,54] led to stability, we likewise expect that the intermittent synchronization obtained in our present study leads to stability of the driven oscillations, as would be indicated by a stability analysis of the non-autonomous one-dimensional differential equation

$$\frac{d\phi }{dt}=\omega_{2}+q_{t}(\phi_{1(0)}+\omega_{1}t,\phi ),$$

where *ϕ*(*t*) is the phase of the driven oscillator and *ϕ*_1(0)_ + *ω*_1_*t* is the phase of the driving oscillator.

## 8. Conclusion

Interacting dynamical systems can have time-varying coupling functions, where the net coupling strength depends in a number of different ways on the time, sometimes resulting in synchronization transitions. The experiments on the cardiorespiratory and neural delta-alpha coupling functions whose results are shown in figure 1, for example, illustrate the existence of time-varying functional relationships that can cause synchronization transitions.

A model of two coupled oscillators with time-evolving coupling functions has been shown to exhibit transitions to/from synchronization even when the net coupling strength remains constant. The analysis was carried out in terms of the phase difference between the oscillators. The corresponding numerical simulations show that, in the case of time-varying coupling functions, one can have sequential epochs of synchrony and asynchrony while the net coupling strength remains unchanged. Thus, by itself, the net coupling strength does not provide enough information to describe the dynamics of the interacting systems. To generalize the results, based on the model considered, we discussed three main ideas arising from the periodic function *f*(*t*). The first of these was that, when *φ*(*t*): = *f*(*t*)*t* varies slowly with time, the dynamics of the two coupled oscillators induces synchrony over the slow timescale. The second was that when, by contrast, *φ*(*t*) has rapid angular velocity, the oscillators do not synchronize. The third observation was the combined effect: the oscillators can exhibit sudden changes between synchrony and drifting phase difference occurring at transitions between slow variation and fast winding of *φ*(*t*). So we have transitions in exhibited behaviour due to time-variability of the coupling functions despite constant net coupling strength. This confirms that, in the time-variable setting, the net coupling strength does not give sufficient information about the interaction of the oscillators to predict their behaviour.

Note that this insufficiency of the net coupling strength as a criterion carries implications for information-theoretic methods that assess the statistical mutual dependences of signals from interacting systems [10,34–36,58,59]. The latter are statistical measures that can determine a causal relationship and the predominant direction of influence, thus measuring a directed functional connectivity. In this way, however, they usually reveal only the net coupling strength and direction, but are unable to detect variations in sub-coupling components like those used in the present study.

## Data accessibility

Data used for analysis of coupling functions shown in figure 1 were collected during the project BRACCIA https://cordis.europa.eu/project/rcn/74753/factsheet/en and are available upon request to A.S. Codes used to generate data in figures 2 and 3 are also available.
